# Parent-Reported Health-Related Quality of Life (HRQoL) of NICU Graduates in Their First Year: A Prospective Cohort Study

**DOI:** 10.3390/ijerph22030447

**Published:** 2025-03-17

**Authors:** Parel Heuvink, Nienke H. van Dokkum, Koenraad N. J. A. Van Braeckel, Helene A. Bouma, Karianne E. Kraft, Arend F. Bos, Paul F. M. Krabbe

**Affiliations:** 1 Division of Neonatology, Department of Pediatrics, Beatrix Children’s Hospital, University Medical Center Groningen, University of Groningen, Hanzeplein 1, P.O. Box 30.001, 9713 GZ Groningen, The Netherlands; p.heuvink@student.rug.nl (P.H.); schoensel@yahoo.com (K.N.J.A.V.B.); h.a.bouma@umcg.nl (H.A.B.); k.e.kraft@umcg.nl (K.E.K.); a.f.bos@umcg.nl (A.F.B.); 2 Department of Epidemiology, University Medical Center Groningen, University of Groningen, Hanzeplein 1, P.O. Box 30.001, 9713 GZ Groningen, The Netherlands; p.f.m.krabbe@umcg.nl

**Keywords:** health-related quality of life, NICU graduates, parental perspective

## Abstract

Health-related quality of life (HRQoL) in neonatal intensive care unit (NICU) graduates during the first year after birth is unknown. Gaining more insight into parental perspectives on HRQoL in this group may aid healthcare professionals in follow-up care. We aimed to assess HRQoL of NICU graduates during their first year after birth from a parental perspective using the newly developed Infant Quality of Life Instrument mobile application questionnaire. This was a prospective cohort study including NICU graduates of all gestational ages (N = 108). We assessed which of seven HRQoL domains, Sleeping, Feeding, Breathing, Stooling, Mood, Skin, and Interaction, proved most problematic during infants’ first year after birth and whether there were differences between the gestational age groups. The three domains proving most problematic from the parents’ perspective were Feeding (ranging from 14% to 43%), Sleeping (ranging from 23% to 42%), and Interaction (decreasing from 86% to 19%). The trajectories of extremely preterm infants were more frequently problematic than those of other groups. Healthcare professionals should focus on these most problematic domains in their follow-up care.

## 1. Background

Despite its life-saving raison d’être, a neonatal intensive care unit (NICU) is a stressful environment for both infants and parents [1,2]. Stressors include noise, light, handling, painful procedures, and parental separation [2]. This is the case for both preterm infants and full-term infants with a difficult start in life. Even though the survival rate of preterm infants has increased in recent decades, only 50% of infants born at 24 weeks survive the neonatal period [3]. Short-term and long-term morbidities still remain increased in preterm infants, with high incidences of bronchopulmonary dysplasia (BPD), necrotizing enterocolitis (NEC), and retinopathy of prematurity [4]. Moreover, compared with their full-term peers, preterm infants are more likely to develop conditions such as cerebral palsy or epilepsy and experience cognitive [5] and motor problems throughout childhood [6], possibly persisting into adolescence [5]. Full-term infants may be admitted to the NICU on account of respiratory insufficiency, infections, asphyxia, or congenital abnormalities [7]. In the long term, perinatal asphyxia can lead to neurodevelopmental impairments, cerebral palsy, and loss of hearing or vision [7]. Children with congenital cardiac abnormalities are reported to be at greater risk of developing feeding problems [8] or executive function deficits [9], and they score lower in expressive language tasks than their peers [10].

During the first years of life, infants who have been admitted to the NICU often have mild to severe health problems. These problems can be quantified using a health-related quality of life (HRQoL) score. Studies on HRQoL in school-aged children and young adults report poorer scores among preterm-born children compared to healthy full-term-born children [11,12] or no differences with peers [13]. Full-term NICU graduates may also face problems after staying in hospital. For example, children with congenital cardiac disease have been reported to experience a lower HRQoL than their healthy peers [14]. There is ample research on HRQoL between childhood and adulthood mainly in those born preterm. However, research on early HRQoL, particularly in the first year of life, is scarce because instruments measuring HRQoL this early are unavailable. The most commonly used instrument (i.e., the PEDSQL questionnaire) is designed to capture HRQoL from 2 years of age onwards. Thus, the HRQoL in the first year after birth is not well known. In this early period, HRQoL may include regulatory problems such as feeding, sleeping, and fussing/crying. It has been reported that preterm infants are at greater risk of developing regulatory problems during the first 18 months after birth compared to healthy full-term infants [15].

Recently, a new instrument for measuring HRQoL was developed, the Infant Quality of Life Instrument (IQI)—the first generic, preference-based HRQoL application appropriate for children in this age group. This instrument assesses seven HRQoL domains: Sleeping, Feeding, Breathing, Stooling, Mood, Skin, and Interaction [16]. It provides healthcare professionals with insight into parents’ perspectives on problematic HRQoL domains. Next, they can use this information to devise and implement suitable early interventions. We aimed to investigate parent-reported HRQoL of NICU graduates during their first year after birth.

## 2. Methods

### 2.1. Setting and Population

This was a prospective cohort study including infants who had been admitted to the NICU at the University Medical Center Groningen for at least four days between 1 January 2019 and 31 December 2019. The infants’ parents provided written informed consent to participate in the study, either during their infants’ NICU stay or by e-mail after discharge if consent could not be obtained during NICU admission. After providing consent, parents received an e-mail link that allowed them to access the IQI mobile application. We asked parents to complete the IQI during their infant’s NICU stay (if applicable) and again at 3, 6, 9, and 12 months after birth. For these time points, we sent the parents reminder e-mails. In the case of prematurity, the reminders were sent to align with the infants’ corrected ages. Beyond the instructions provided in the IQI, we did not provide additional instructions to parents. The institutional review board (the medical ethical committee) approved the study (METc 2019-017). The study was not registered online in a clinical trial registry.

### 2.2. The Infant Quality of Life Instrument (IQI)

**Figure 1:**
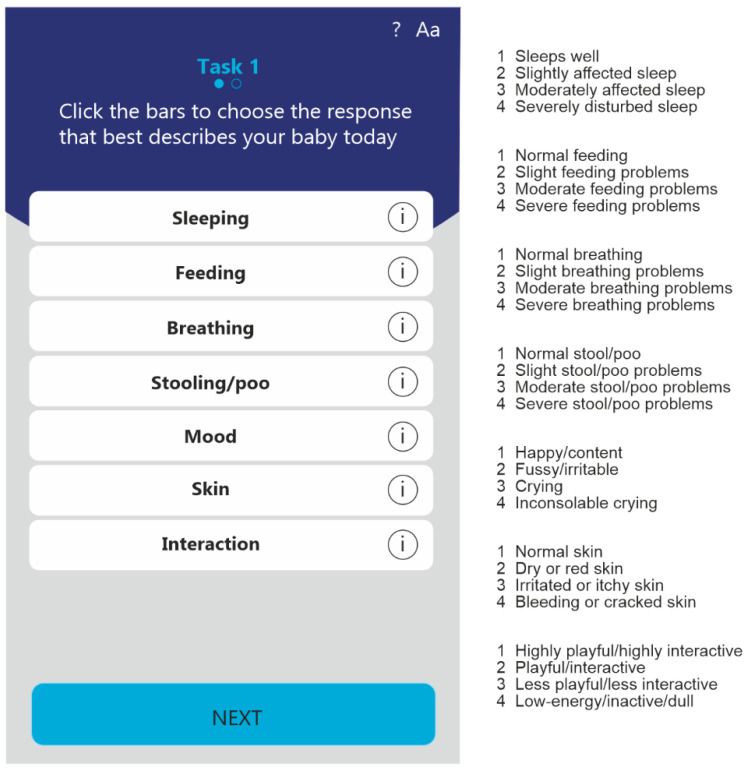
Example of the infant quality of life instrument (IQI) and the health-related quality of life (HRQoL) domains and levels.

The IQI assesses seven domains: Sleeping, Feeding, Breathing, Stooling, Mood, Skin, and Interaction. Each domain is allocated a score on a 4-point scale, a score of 1 indicating no problems in that domain to a score of 4 indicating severe problems in that domain. Although the IQI was previously designed to assess HRQoL, it was not specifically designed for this study [17]. We show an example of the IQI mobile application and the HRQoL domains and their levels in Figure 1. Each domain represents a specific HRQoL domain for infants that was selected based on literature searches that were subsequently evaluated by pediatric specialists. After this evaluation, parents were asked to also evaluate the domains and provided feedback on the domains proposed. Parents were also requested to rank the domains in order of importance, leading to the eventual seven domains of HRQoL presented in the IQI. This IQI was developed in 2018 and designed to calculate an overall HRQoL score, which can be calculated when the sample size is sufficient to do so. In this study, that was not the case, therefore we used the IQI to descriptively evaluate the underlying seven domains of HRQoL.

### 2.3. Data and Statistical Analysis

First, we described background characteristics of our study population using descriptive statistics. Second, we calculated frequencies of reported problems across domains and time points, as well as different background characteristics, and tested these to determine whether there were differences by applying a chi-square test for trends using SPSS Statistics for Windows, Version 27 (IBM Corp, Armonk, NY, USA). Third, we constructed trajectories per HRQoL domain and tested differences in trajectories across gestational age groups with multilevel modeling using MLWiN (University of Bristol, Bristol, UK, Version 3.05). We built seven separate models, one for each HRQoL domain. For each domain, the specific time points were nested within gestational age groups. The advantage of multilevel modeling over traditional measures, such as a repeated measures analysis of variance, is that multilevel analysis calculates weighted means and their standard errors accounting for the number of data points per unique participant. Particularly for our population with missing data over time, this approach allowed us to include all responses of participants, with more weight to the mean for respondents with more data points compared to those with fewer data points. We did not impute missing values, as we wanted the sample to reflect clinical practice. In multilevel modeling, a *t*-test is used to assess differences between an estimated mean and the intercept (with the full-term group and completed by parents during the NICU stay as the reference), while a chi-square test with one degree of freedom is used to assess differences between two estimated means.

A *p* value of 0.05 or less was considered statistically significant in all tests and models.

## 3. Results

### 3.1. Background Characteristics

In the first year of inclusion, there were 311 admissions to our NICU. Of those, 233 were eligible because they were admitted for more than four days. In total, 188 parents received account information to participate in completing the IQI. The difference is caused by logistical reasons, such as insufficient language skills, lack of an available e-mail address, death, no permission to participate, or no reaction to our invitation to participate. Finally, 108 parents completed the IQI at least once. That makes our response rate 46% of all eligible infants, or 57% of all participating parents.

In total, the IQI was completed 219 times by the 108 parents, including 31 (29%) parents of full-term children, 19 (18%) of moderately late preterm children (gestational age 32–37 weeks), 45 (42%) of very preterm children (gestational age 28–32 weeks), and 13 (12%) of extremely preterm children (gestational age < 28 weeks; see Table 1). Approximately one-third of the data (31.7%) were collected at 3 months (corrected) age, and one-fifth (19.9%) during the NICU stay. Most parents completed the IQI only once.

### 3.2. Parent-Reported Problems During the First Year After Birth

Overall, parents most frequently reported problems in the HRQoL domains Feeding, Sleeping, and Interaction (Table 2). For Feeding and Interaction, the frequency of problems was highest during the NICU stay, while Sleeping problems were more frequently reported after discharge. Feeding problems were most frequently reported as mild, but some parents reported severe problems. Sleeping problems remained relatively frequent during the first year after birth (23% to 42%), and most problems were reported as being mild. For all groups the frequency of problems with Interaction decreased after discharge from the NICU. While problems concerning the domains Breathing and Skin were frequently reported during infants’ NICU stay, few parents reported problems in these two domains after discharge. Additionally, reported problems for Breathing and Skin were milder than for Feeding, Sleeping, and Interaction. Stooling problems were reported during the entire first year but were considered mild. Mood problems were infrequent and mild during the first year after birth. When comparing the domains by background characteristic (Table 3), males overall have more breathing problems and parents of first-born children report more interaction problems across time points.

### 3.3. Trajectories Across Gestational Age Groups

**Figure 2:**
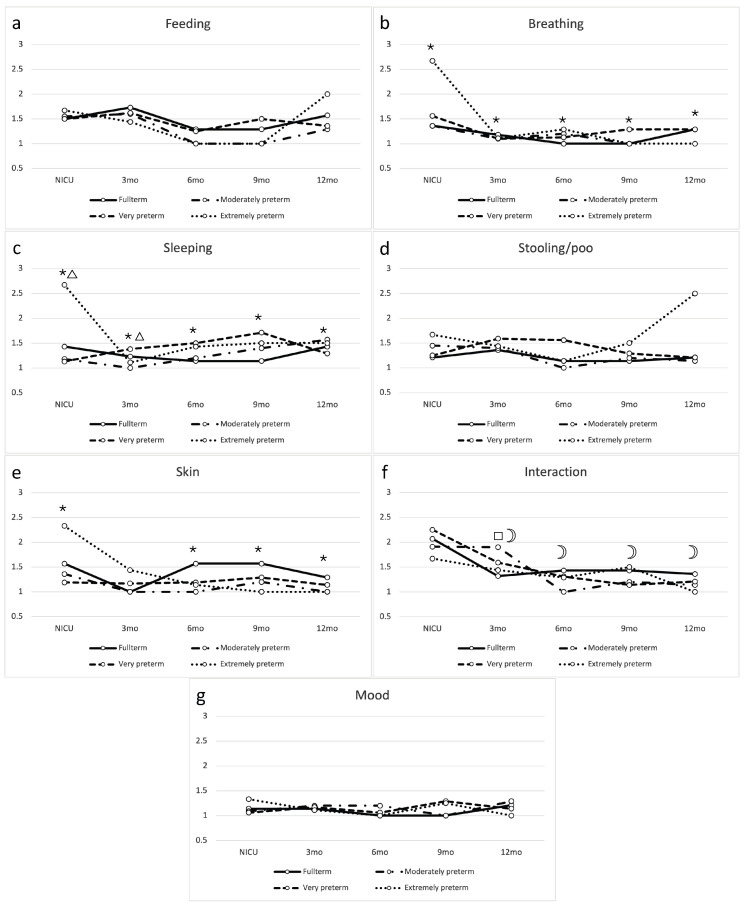
Trajectories of the seven domains of health-related quality of life (HRQoL) for all time points and gestational age groups, (**a**) Feeding, (**b**) Breathing, (**c**) Sleeping, (**d**) Stooling/poo, (**e**) Skin, (**f**) Interaction and (**g**) Mood. On the y-axis, the average scores for HRQoL are presented. The symbols ☽, □, △, and * are used to indicate significant differences resulting from a *t*-test in the gestational age groups: full-term, moderately preterm, very preterm, and extremely preterm, respectively, when compared to the reference group. The reference group is the full-term population during their neonatal intensive care unit (NICU) stay.

For Feeding, there was no significant difference between the trajectories of the gestational age groups (Figure 2a). Problems reported for Breathing were mild, but parents of extremely preterm children reported significantly more breathing problems across time points (*p* values between <0.001 and 0.015, Figure 2b). Parents of extremely preterm children also reported more sleeping problems across time points, but particularly during NICU stay (*p* values between 0.001 and 0.050, Figure 2c). There were no significant changes in problems over time for Stooling, nor were there differences between gestational age groups (Figure 2d). In all groups, problems concerning the domain Skin seemed to decrease after discharge from the NICU. For very preterm children the course of skin problems was significantly different only for the first three months after birth (*p* = 0.006). The course of skin problems for extremely preterm children was significantly different for all time points except for three months corrected age (*p* between 0.001 and 0.327, Figure 2e). There were no differences between gestational age groups for the domain Interaction (Figure 2f). Nearly all infants, irrespective of their gestational age, were reported to be happy/content at all time points. There were no statistically significant differences between the trajectories of the groups for the domain Mood (Figure 2g).

## 4. Discussion

This study on HRQoL demonstrated that parents of NICU graduates report Interaction, Sleeping, and Feeding as the most problematic domains during the first year of life. If present, reported problems were generally mild, although sometimes, severe problems were reported for Feeding. There were small differences between the gestational age groups trajectories, except for Breathing and Sleeping in extremely preterm infants particularly during their NICU stay.

We found that parents report Feeding, Sleeping, and Interaction as the most problematic domains. For feeding, this is in line with other studies describing that NICU graduates have a higher prevalence of feeding problems between the ages of 1 and 2 years, without differences between gestational age groups [18]. Many infants admitted to a NICU require oral/nasal gavage feeding instead of being breast-fed or bottle-fed, and we hypothesize that this could influence the score allocated to Feeding, also after discharge. For Sleeping, some studies report preterm infants as having more problems than full-term infants, but these studies only included infants from the age of one year after birth [19,20,21]. We hypothesize that the quality of sleep of infants may be poorer because of the increased stress levels during NICU stay [22], or the increased prevalence of gastroesophageal reflux and obstructive sleep apnea in preterm infants [23]. For Interaction, some studies found that attachment scores between parents and preterm infants are lower than those between parents and full-term infants [24], but we found no studies investigating the course of attachment over time. The reason why more problems in Interaction are reported during the NICU stay and shortly thereafter may be due to a disturbed attachment between infants and parents during the NICU period. This is supported by research reporting that parents of preterm NICU graduates experience more symptoms related to depression and anxiety [25] and increased levels of stress up to one year after birth [26]. Thus, from the parents’ perspective, staying in a NICU may predispose infants to problems in these three domains.

For the domains Sleeping and Breathing, we found poorer scores for extremely preterm infants during NICU stay than other gestational age groups. For Sleeping, we hypothesized that this is the result of frequent invasive procedures and the NICU environment that these children are exposed to for a longer period. Studies investigating sleep during NICU stay and after discharge from the NICU often do not differentiate between gestational age groups [19,20,21]. Regarding Breathing, extremely preterm infants depended more on invasive ventilatory support than other gestational age groups, which could explain why parents report this domain as problematic more often. With the elimination of ventilatory support after discharge, we could not detect any differences between gestational age groups anymore. This seems to be in accordance with studies investigating children at older ages [27].

Regarding the domain Skin, we found a different trajectory for extremely and very preterm infants only. The skin of very preterm infants may be underdeveloped and therefore the infants may have had more skin problems during the first weeks and months after birth. From a nursing perspective, skin care should therefore receive sufficient attention, and parents may be trained in recognizing skin problems in their children.

General HRQoL after discharge from the NICU seemed to improve for some domains, while the problems in other domains remained constant. From the parental perspective, differences in trajectories between gestational age groups can be explained largely by extremely preterm infants’ experiencing more health-related issues during their NICU stay. After 3 months (corrected) age, differences between gestational age groups no longer existed. Apparently, for the first year after birth, the number of days spent in a NICU was less important for HRQoL than the fact whether the infant had stayed in a NICU at all. Nevertheless, the parental perspective regarding HRQoL during the first year after birth, as reflected in percentages of reported problems, is important enough to take into consideration in follow-up care.

HRQoL may depend on the perspective of the assessor. For example, a systematic review by Vieira et al. that included both NICU graduates and their parents, reported that parents assessed HRQoL lower than their children [28]. Adams et al. reported that parents differ from neonatologists regarding their perspective on HRQoL [29]. We examined the important parental perspective, and we know that most parents are able to reliably assess their children’s HRQoL [30]. Different scores may have been obtained had the children been assessed by physicians instead of their parents. However, seeing that infants spend almost the entire day with their parents, we believe that the parental perspective is invaluable. When interpreting these results, it is therefore important to keep in mind that they are based solely on information obtained from the parental perspective.

### 4.1. Strengths and Limitations

Our study is the first to assess HRQoL in infants during the first year after birth in a systematic manner with a short and easy to complete questionnaire. Second, our choice of multilevel modeling allowed us to minimize the bias of missing data. A limitation to our study is the small amount of data at some time points. This also hampered our ability to perform in-depth analyses of neonatal morbidities. Even though all parents were informed that the instrument was meant to be completed five times during the first year of life and received a reminder at those time points, few parents completed the IQI more than once. Low response rates may be a result of post-traumatic stress symptoms that these parents often experience [26]. Additionally, the method of invitation, i.e., e-mail, may not be sufficient to reach parents after they have been discharged from the NICU. A second limitation is the lack of a healthy full-term control group. We also recognize that this is a single center study in a level III–IV NICU, which may limit the generalizability of our results to other centers.

### 4.2. Implications

The current study may serve as a first step towards devising and implementing interventions during NICU stay that focus on parental perspectives of infant cues, particularly in the domain Sleeping for extremely preterm infants, and for Interaction. Potential interventions may include parental education on the possible HRQoL problems that may be encountered after NICU discharge, and what parents may be able to do themselves. Additionally, in targeted neonatal follow-up programs, the IQI may be implemented as a screening tool to aid healthcare professionals in tailoring their care for infants. The next step in research should be to gain insight into what exactly parents experience as problematic when they assess the different domains. The IQI does not provide such information, which could be examined in future research, for example, through qualitative interview studies. It may also be interesting to explore HRQoL in specific NICU graduates, such as those with NEC and BPD. This may eventually lead to altered neonatal follow-up guidelines that also include HRQoL aspects, in addition to neurodevelopment outcomes.

## 5. Conclusions

Regarding HRQoL, we found that parents of NICU graduates report most problems in the domains Feeding, Sleeping, and Interaction during the first year after birth. While there are few differences in trajectories across gestational age groups, parents of extremely preterm infants reported more problems in Sleeping, Breathing, and Skin domains, particularly during the NICU stay. Integrating parental perspectives on HRQoL may improve neonatal and follow-up care for NICU graduates. We advise healthcare professionals to incorporate HRQoL assessments into neonatal follow-up care. Future studies should focus on the effect of separate neonatal morbidities and further investigate parental perspectives in greater depth.

## Figures and Tables

**Table 1:** Background characteristics (N = 108).

	Full-Term (n = 31)	Moderately Late Preterm (n = 19)	Very Preterm (n = 45)	Extremely Preterm (n = 13)
Gestational age (weeks)	38 + 4 (SD 1.26)	34 + 0 (SD 1.52)	30 + 0 (SD 1.07)	26 + 2 (SD 0.93)
Birth weight (grams)	3489 (SD 629.45)	2295 (SD 543.27)	1468 (SD 288.22)	960 (SD 155.41)
Sex				
Male	17 (55%)	10 (53%)	26 (58%)	9 (69%)
Female	14 (45%)	9 (47%)	19 (42%)	4 (31%)
Multiple birth				
Yes	0 (0%)	2 (11%)	14 (31%	1 (8%)
No	31 (100%)	17 (89%)	31 (69%)	12 (92%)
Firstborn child				
Yes	12 (39%)	7 (37%)	32 (71%)	9 (69%)
No	17 (55%)	9 (47%)	12 (27%)	4 (31%)
Surgery				
None	23 (74%)	13 (68%)	45 (100%)	11 (85%)
GI tract	3 (10%)	5 (26%)	0 (0%)	2 (15%)
Cardiac	3 (10%)	1 (5%)	0 (0%)	0 (0%)
Airway	1 (3%)	0 (0%)	0 (0%)	0 (0%)
Urology	1 (3%)	0 (0%)	0 (0%)	0 (0%)
Ventilation support				
None	4 (13%)	2 (11%)	1 (2%)	0 (0%)
High flow/low flow	1 (3%)	0 (0%)	2 (4%)	1 (8%)
CPAP	3 (10%)	6 (32%)	28 (62%)	3 (23%)
NIVM	0 (0%)	0 (0%)	0 (0%)	2 (15%)
Conventional	21 (68%)	11 (58%)	14 (31%)	5 (38%)
HFO	2 (6%)	0 (0%)	0 (0%)	2 (15%)
Total completed questionnaires	67	38	89	25
Number of completed HRQoL app questionnaire				
1×	12 (39%)	7 (37%)	19 (42%)	6 (46%)
2×	10 (32%)	6 (31%)	14 (31%)	3 (23%)
3×	3 (10%)	5 (26%)	6 (13%)	3 (23%)
4×	4 (13%)	1 (5%)	6 (13%)	1 (8%)
5×	2 (6%)	0 (0%)	0 (0%)	0 (0%)
Timing of completed HRQoL app questionnaires				
During NICU	14 (21%)	11 (29%)	16 (18%)	3 (12%)
3 mo	22 (33%)	10 (26%)	29 (33%)	9 (36%)
6 mo	7 (10%)	5 (13%)	16 (18%)	7 (28%)
9 mo	10 (15%)	5 (13%)	14 (16%)	4 (16%)
12 mo	14 (21%)	7 (18%)	14 (16%)	2 (8%)

Abbreviations: SD, standard deviation; NICU, neonatal intensive care unit; GI tract, gastrointestinal tract; CPAP, continuous positive airway pressure; NIMV, nasal intermittent mandatory ventilation; HFO, high frequency oscillation; 3 mo, 3 months (corrected) age; 6 mo, 6 months (corrected) age; 9 mo, 9 months (corrected) age; 12 mo, 12 months (corrected) age.

**Table 2:** Reported frequency of a problem in the total sample by time point.

	**NICU (n = 44)**	**3 mo (n = 70)**	**6 mo (n = 35)**	**9 mo (n = 33)**	**12 mo (n = 37)**	***p* Value**
Feeding	19 (43%)	27 (39%)	5 (14%)	6 (18%)	12 (32%)	0.046
Breathing	18 (41%)	7 (10%)	5 (14%)	4 (12%)	7 (19%)	0.052
Sleeping	12 (27%)	16 (23%)	12 (34%)	14 (42%)	11 (30%)	0.233
Stooling/poo	11 (25%)	27 (39%)	9 (26%)	8 (24%)	8 (22%)	0.264
Skin	18 (41%)	7 (10%)	7 (20%)	6 (18%)	6 (16%)	0.071
Interaction	38 (86%)	37 (53%)	10 (29%)	8 (24%)	7 (19%)	<0.001
Mood	4 (9%)	9 (13%)	2 (6%)	5 (15%)	6 (16%)	0.359

NICU, neonatal intensive care unit; 3 mo, 3 months (corrected) age; 6 mo, 6 months (corrected) age; 9 mo, 9 months (corrected) age; 12 mo, 12 months (corrected) age.

**Table 3:** Reported frequency of a problem for different background characteristics.

	**Sex (Male vs. Female)**	**Multiple Birth (No vs. Yes)**	**First Born (No vs. Yes)**	**Surgery (No vs. Yes)**	**Ventilation Support (No vs. Yes)**
Feeding	41 (32%) vs. 28 (31%)	60 (31%) vs. 9 (33%)	26 (33%) vs. 42 (33%)	58 (31%) vs. 11 (31%)	3 (18%) vs. 66 (33%)
Breathing	30 (23%) vs. 11 (12%) *	39 (20%) vs. 2 (7%)	11 (14%) vs. 27 (21%)	34 (18%) vs. 7 (20%)	3 (18%) vs. 38 (19%)
Sleeping	37 (29%) vs. 28 (31%)	55 (29%) vs. 10 (37%)	26 (33%) vs. 36 (29%)	56 (30%) vs. 9 (26%)	3 (8%) vs. 62 (31%)
Stooling/poo	32 (25%) vs. 31 (34%)	53 (28%) vs. 10 (37%)	23 (29%) vs. 39 (31%)	54 (29%) vs. 9 (26%)	6 (35%) vs. 57 (28%)
Skin	30 (23%) vs. 14 (15%)	42 (22%) vs. 2 (7%)	12 (15%) vs. 29 (23%)	36 (20%) vs. 8 (23%)	3 (18%) vs. 41 (20%)
Interaction	56 (44%) vs. 44 (48%)	89 (46%) vs. 11 (41%)	27 (34%) vs. 3 (50%) *	86 (47%) vs. 14 (40%)	7 (41%) vs. 93 (46%)
Mood	14 (11%) vs. 12 (13%)	23 (12%) vs. 3 (11%)	9 (11%) vs. 16 (13%)	20 (11%) vs. 6 (17%)	1 (6%) vs. 25 (12%)

* *p* < 0.05.

## Data Availability

Deidentified individual participant data will be made available upon reasonable request.

## Abbreviations

BPD, bronchopulmonary dysplasia; HRQoL, health-related quality of life; IQI, Infant Quality of Life Instrument; NEC, necrotizing enterocolitis; NICU, neonatal intensive care unit; UMCG, University Medical Center Groningen.
